# Efficacy of the acupressure wrist-ankle strap in mild insomnia patients with anxiety disorders: study protocol for a randomized controlled trial

**DOI:** 10.1186/s13063-021-05725-4

**Published:** 2021-11-04

**Authors:** Ying Yuan, Qinghui Zhou, Fanfu Fang, Weihong Li, Yanli You

**Affiliations:** 1 Department of Traditional Chinese Medicine, Naval Medical University, No. 800 Xiang Ying Road, Yangpu District, Shanghai, 200433 China; 2 Department of Acupuncture and Moxibustion, the First Affiliated Hospital of Naval Medical University, No. 168 Chang Hai Road, Yangpu District, Shanghai 200433 China; 3 Department of Rehabilitation Medicine, the First Affiliated Hospital of Naval Medical University, No. 168 Chang Hai Road, Yangpu District, Shanghai 200433 China

**Keywords:** Anxiety, Comorbid insomnia, Acupressure wrist-ankle strap, HPA axis, RCT, Study protocol

## Abstract

**Background:**

Insomnia is very common in current society, and patients are often accompanied by a certain degree of anxiety, depression, etc. Recent studies have found that the hypothalamic-pituitary-adrenal (HPA) axis excitement-inhibition state is an important indicator of sleep quality. Wrist-ankle acupuncture (WAA) is safe and effective for insomnia. Based on WAA theory, the acupressure wrist-ankle straps are portable WAA point compression straps that can treat diseases by automatically applying pressure to the treatment location and being operated by patients themselves. We design this trial to evaluate the clinical effect of the acupressure wrist-ankle strap in the treatment of mild insomnia patients with anxiety disorders.

**Methods/design:**

This trial is a parallel-design, patients-assessor blinded, randomized, sham-controlled. In total, 114 patients diagnosed with mild insomnia and anxiety disorders will be randomly assigned into two groups, the acupressure wrist-ankle strap group or the non-acupressure wrist-ankle strap group; they will receive treatments for eight weeks with five sessions each week. Rating scales, sleep monitors, and laboratory tests will be used to observe the clinical effect. From the perspective of the circadian secretion of peripheral blood-related hormones in the hypothalamic-pituitary-adrenal (HPA) axis, the possible mechanism of acupressure wrist-ankle straps for treating insomnia is studied.

**Discussion:**

The results of this study will confirm the efficacy of acupressure wrist-ankle strap in treating mild insomnia patients with anxiety disorder and whether its mechanism is related to the HPA axis. The acupressure wrist-ankle strap may become a pure physical, no side effect treatment of mild insomnia.

**Trial registration:**

Chinese Clinical Trial Registry ChiCTR2000039352. Registered on 24 October 2020.

## Background

After a lapse of 9 years, the British Psychopharmacological Association (BAP) updated its consensus on treating insomnia, parasomnia, and circadian rhythm disorders in 2019. The consensus mentioned that about half of all patients with diagnosed insomnia have at least one comorbidity of mental disorder [1]. Insomnia patients are often accompanied by a certain degree of anxiety, depression, and other bad moods. The specific manifestations of insomnia patients with an anxiety disorder include insomnia with significant anxiety symptoms or emotional disorders such as nervousness, worry, and irritability [2]. Clinical studies found that about 50% of insomnia patients are accompanied by mental disorders [3, 4]. Compared with non-anxiety insomnia patients, insomnia patients with anxiety have significantly reduced sleep efficiency, total sleep time, and percentage of slow-wave sleep, which significantly reduces patients’ quality of life and brings severe social dysfunction and economic burden [5, 6].

Although the pathogenesis of insomnia has not yet been fully understood, studies [7] have found that the excitation-inhibition state of the hypothalamic-pituitary-adrenal (HPA) axis is an important indicator of sleep quality. The HPA axis is mainly composed of the hypothalamus, pituitary gland, and adrenal glands, and its activity is regulated by corticotrophin-releasing hormone (CRH). CRH promotes the secretion of adreno-cortico-tropic-hormone (ACTH) from the pituitary. ACTH is released into the blood and reaches the adrenal gland through systemic circulation, prompting the adrenal cortex to release glucocorticoids and cortisol (CORT). ACTH can improve the irritability of the cerebral cortex and is an integral part of the sleep-wake regulation mechanism. Many monoamine neurotransmitters, such as serotonin (5-HT) and norepinephrine (NE), also play an essential role in the regulation of the HPA axis [8].

At present, hypnotics recommended by the guidelines for insomnia include benzodiazepine receptor agonists (BZRA) (including non-benzodiazepines and benzodiazepines) or melatonin receptor agonists (e.g., ramelteon), sedative antidepressants (trazodone, mirtazapine, fluvoxamine, and doxepin). The latter is especially suitable for insomnia patients with depression or anxiety or both of them. However, patients may have dizziness, drowsiness, cognitive dysfunction, apnea, and other adverse reactions after taking such drugs. In addition to own adverse drug reactions, there were also insomnia rebound and withdrawal reactions [1, 3, 9]. Patients with mild insomnia have occasional insomnia, which often has little effect on work, and often does not require drug treatment. It can be improved by non-drug treatment represented by sleep hygiene education and cognitive behavioral therapy for insomnia (CBTI) [10], and most of them are against medication. Therefore, it is of great significance for patients with mild insomnia to develop a purely physical, easy-to-operate, non-invasive, non-toxic, and side-effect and repeated use of drug replacement therapy.

Wrist-ankle acupuncture (WAA) is a unique acupuncture therapy performed through the subcutaneous insertion of needles at points on the wrist and ankle regions [11]. WAA does not induce pain or “needling sensation.” The acupressure wrist-ankle strap (China patent ZL201420475426.8) is a device developed based on WAA therapy. Unlike WAA, the acupressure wrist-ankle strap is non-invasive, recyclable, and does not need an acupuncturist to operate. Patients can wear it on their wrists or ankles like wearing watches. Clinical studies have shown that WAA has a good effect on insomnia [12–14]. However, there is no clinical randomized controlled study to prove the efficacy of the acupressure wrist-ankle strap for insomnia and whether the acupressure wrist-ankle strap and WAA have a similar effect for insomnia. Our clinical experience indicates that patients with mild insomnia can quickly calm down and enter a sleep state after using the strap. If the proposed RCT can verify our hypothesis that acupressure wrist-ankle strap is also effective for insomnia, a new convenient and effective physical therapy is available for insomnia patients with anxiety disorder.

## Methods/design

### Study design

This study is a two-armed, double-blind, randomized, sham-controlled clinical trial aiming to explore the efficacy of the acupressure wrist-ankle strap for insomnia patients with anxiety disorder.

**Fig. 1:**
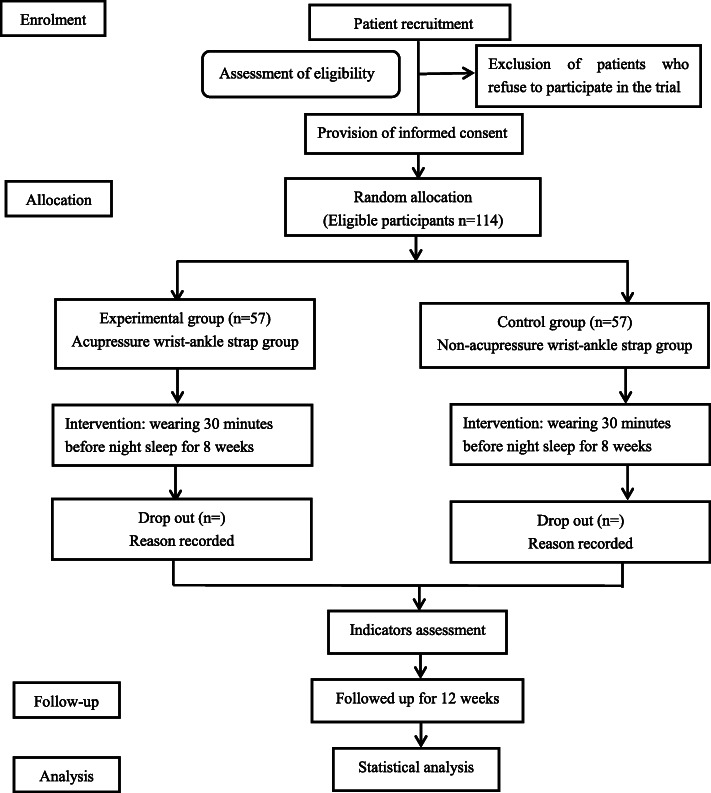
Flowchart of the trial

A total of 114 participants, from the department of acupuncture and moxibustion and the department of internal medicine of traditional Chinese medicine of Shanghai Changhai Hospital, diagnosed with mild insomnia and anxiety disorder following *Guidelines for the Diagnosis and Treatment of Insomnia in China* formulated by the Chinese Sleep Medicine Congress (CSMC, 2017 revised version) [3] and *Expert Consensus on Diagnosis and Treatment of Anxiety and Depression in General Hospitals* formulated by Anxiety disorder cooperation group, Psychiatry Branch, Chinese Medical Association (2012 revised version) [15] will be randomly assigned to two equal groups with 57 patients in each group, including acupressure and non-acupressure wrist-ankle strap group. The outcomes include scales, objective parameters detected by device (wActiSleep-BT Actigraph equipment), and laboratory tests, which will be used to observe the efficacy of the strap on insomnia patients and study its possible mechanism of circadian secretion of related hormones in peripheral blood of hypothalamic-pituitary-adrenal axis (HPA axis). Our study will last 13 weeks in total. The protocol has been registered on the Chinese Clinical Trial Registry (identifier: ChiCTR2000039352) and will be conducted following the Helsinki declaration. This trial will be reported according to the Consolidated Standards of Reporting Trials (CONSORT) statement [16]. The study flowchart is summarized in Fig. 1.

### Participants

Patients meeting the *Guidelines for the Diagnosis and Treatment of Insomnia in China* formulated by CSMC in 2017 and *Expert Consensus on Diagnosis and Treatment of Anxiety and Depression in General Hospitals* formulated by anxiety disorder cooperation group, Psychiatry Branch, CMA in 2012 will be recruited.

#### Inclusion criteria

Eligibility for participation requires each of the following criteria to be met:

- Symptoms comply with both the *Guidelines for the Diagnosis and Treatment of Insomnia in China* formulated by the CSMC in 2017 and *Expert Consensus on Diagnosis and Treatment of Anxiety and Depression in General Hospitals* formulated by anxiety disorder cooperation group, Psychiatry Branch, CMA in 2012
- The occurrence of insomnia was 3 times (including) to 5 times per week
- The Pittsburgh Sleep Quality Index (PSQI) score was 7 to 11 [17], and the Hamilton Anxiety Scale (HAMA) score > 14 [18]
- Age 18–70 years at the time of enrolment (either sex)
- Have not received WAA or other similar treatments
- Prefer physical therapy rather than medication
- Signed informed consent

#### Exclusion criteria

Patients who meet any of the following criteria will be excluded:

- Suffering from insomnia caused by mental diseases or drugs
- Dependence or abuse of alcohol or other substances
- Currently pregnant or lactating
- Having any severe disease of the cardiovascular, liver, kidney, hematopoietic system, and psychiatric disorders
- Allergic to the material of the wrist-ankle straps
- Have taken hypnotic and sedative drugs within one month

#### Dropout criteria

The researcher decides to withdraw from the trial:

- The patient’s condition progressed or continues to deteriorate during the trial, and the clinical trial should be stopped according to the doctor's judgment
- During the trial period, the patient has severe complications or notable physiological changes, and it is not suitable to continue the test
- Serious adverse events occurred during the trial period
- The patient has poor compliance during the trial period, and the treatment dose was less than 80% or more than 120%

Patients quit the trial by themselves:

- During the trial period, patients are unwilling to continue the treatment or take hypnotic-sedative drugs by themselves due to various reasons and actively propose to withdraw from the clinical trial
- Although the patients do not explicitly propose to withdraw from the trial, they might fall out naturally due to loss of follow-up

### Randomization, allocation concealment

A random sequence list is generated using stratified block randomization design and SAS 9.4 (SAS Institute Inc., Cary, NC, USA). The sequence is stratified by gender and age. This trial has an independent statistician assigning the eligible patients to the corresponding intervention code based on the list, who is not be involved in recruitment, implementation, data collection, and data analysis of this trial. All of the random code information is kept by a certain person and stored in sealed opaque envelopes. When an eligible patient is enrolled in the group, the statistician will randomize the patient according to the above method, and the corresponding box with a wrist-ankle strap will be given to the researcher who will be responsible for informing patients the details about the implementation, the wrist-ankle straps used in both groups will have the same packaging. The allocation concealment procedure will not be exposed until the clinical trial is finished.

### Blinding

All the patients, outcome assessors, and data analysts will be blinded to treatment allocation. Every patient of the two groups will receive the same packaging box in the above envelope after entering the group according to the allocation method, which contains the strap (acupressure wrist-ankle strap or non-acupressure wrist-ankle strap) and its instructions, which will guide patients themselves on how to use the straps and informed them related precautions, including the size of the straps, tightness, selection, and adjustment of compression devices and wearing time. To prevent the patients from communicating, we will use follow-up appointments to ensure that the patients will stagger their visits and avoid communication. When the patient withdraws from the trial due to various reasons, emergency unblinding is allowed.

### Interventions

**Fig. 2:**
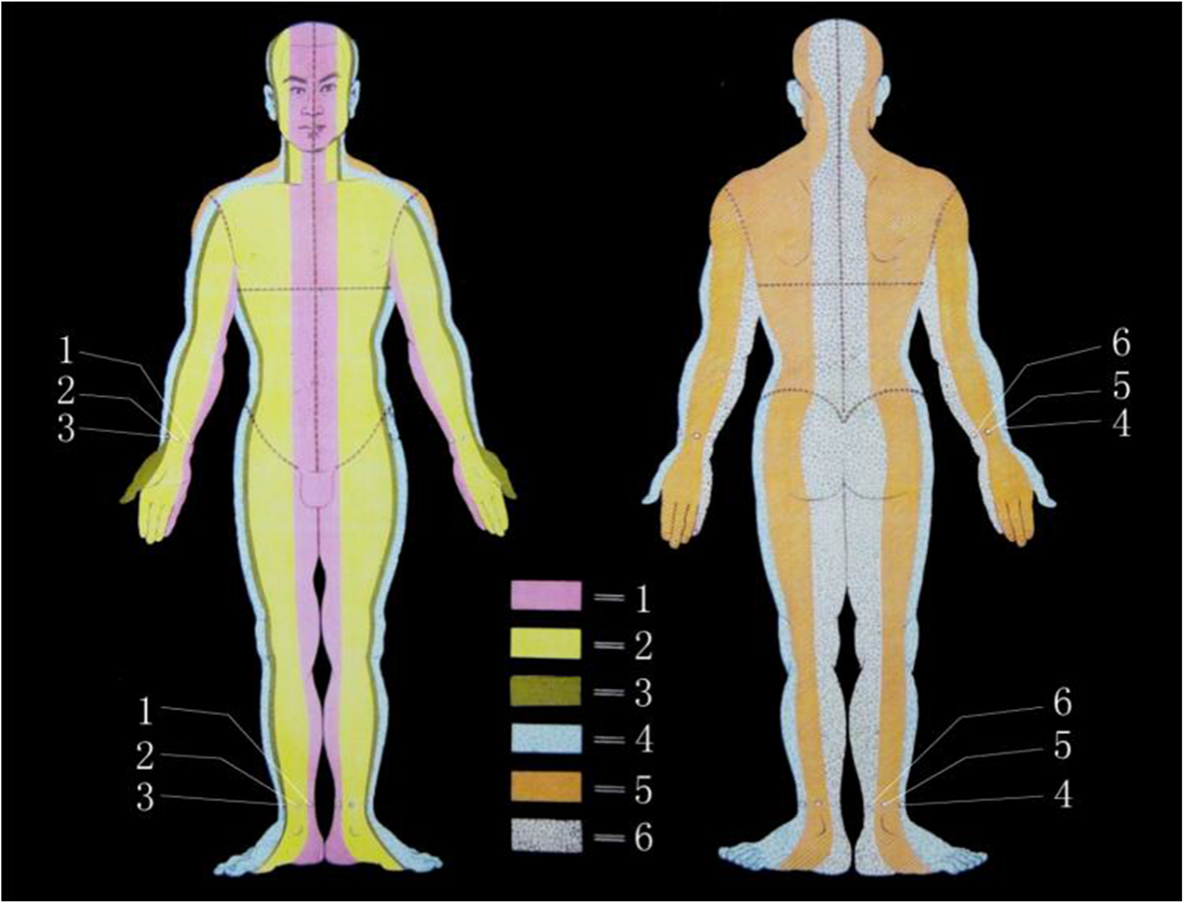
Wrist-ankle acupuncture (WAA) zones and needling points

**Fig. 3:**
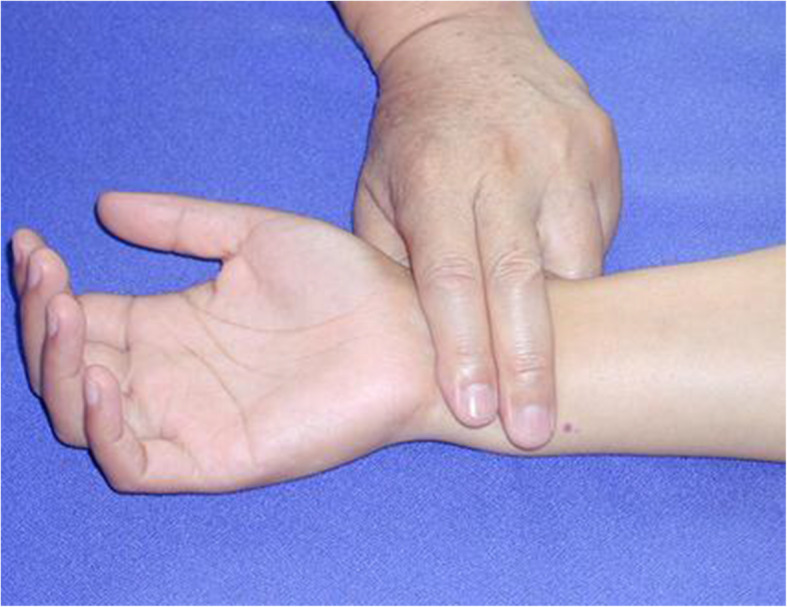
Location of the upper 1 point of wrist-ankle acupuncture (WAA)

Wrist-ankle acupuncture (WAA) is a unique acupuncture method. According to the principle of point selection of WAA, insomnia is a symptom that cannot be located. When doing wrist-ankle acupuncture, we choose upper 1 on both sides [11]. In the acupressure wrist-ankle strap group, the compression component of the wrist-ankle strap compresses upper 1 to produce an acupuncture-like effect. (Figs. 2 and 3)

**Fig. 4:**
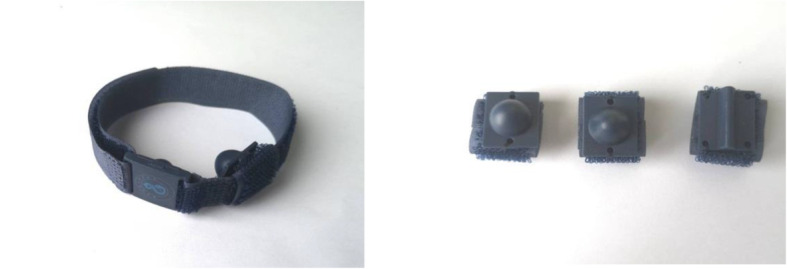
The acupressure wrist-ankle strap and its accessories

The acupressure wrist-ankle strap is a device developed based on WAA therapy (Fig. 4), which is used to replace the invasive operation of WAA. The compression part of the wrist-ankle strap, which is detachable, is used to apply pressure and stimulation to the corresponding WAA point (compression point) to achieve the effect of treating diseases. There is a compression component mounting base on the wrist-ankle strap, and the position can be adjusted according to the needs of the disease, and then 1 to 2 compression components can be installed. Compressing different points can alleviate the pain in different body regions and is usually used for pain, insomnia, motion sickness, seasickness, morning sickness, etc.

#### Acupressure wrist-ankle strap group

**Fig. 5:**
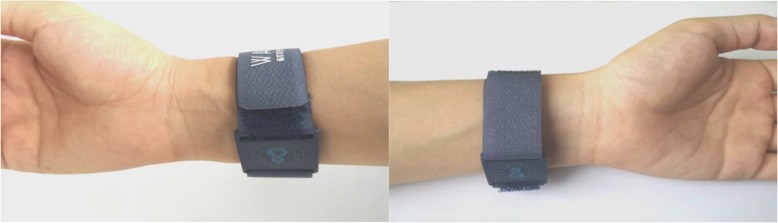
The acupressure wrist-ankle straps on bilateral upper 1

**Fig. 6:**
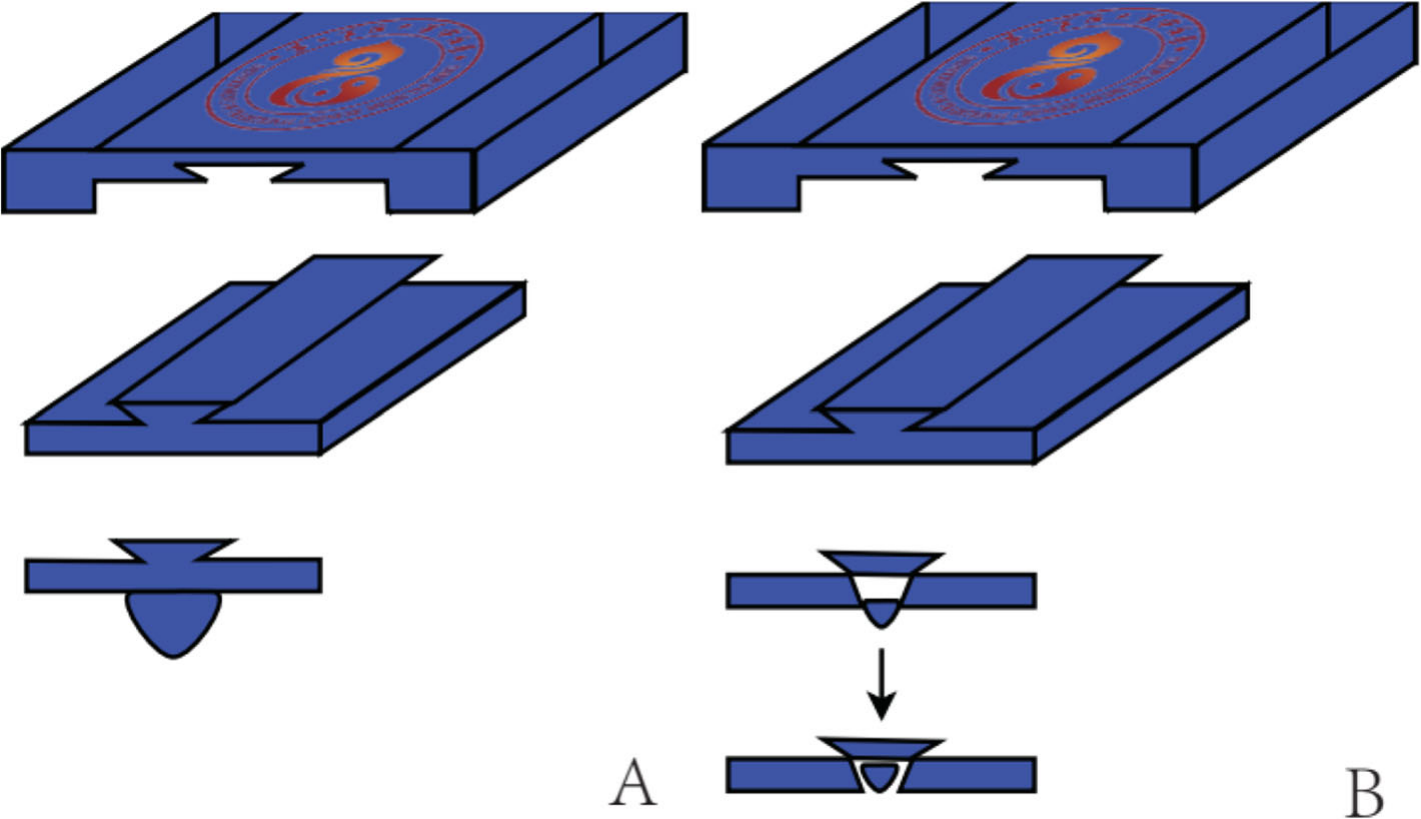
The acupressure wrist-ankle strap compression device is shown in **A**, the non-acupressure wrist-ankle strap compression device is shown in **B**

This group is the intervention group. A portable WAA' point compression therapy device is used, with compression components inside; the bilateral upper 1 are selected as the compression points. The upper 1 is located at the depression between medial border of the ulnar and the tendon of musculus flexor carpi ulnaris, the level of about two fingers above the transverse crease of the wrist. A compression component is installed inside the wrist-ankle strap and worn on both wrists to ensure that the compression component can be compressed to the upper 1 compression point, as shown in Fig. 5. Wear it for 30 min before going to bed every day, and take it off at bedtime, 2 weeks as a course of treatment, four courses in total. The acupressure wrist-ankle strap compression device is shown in Fig. 6A.

#### Non-acupressure wrist-ankle strap group

This group served as the control group. A portable WAA' point compression therapy device is selected. The inner compression component will automatically contract without compression, and it will be worn at the same position on both wrists. Wear it for 30 min before going to bed every day, and take it off at bedtime. 2 weeks as a course of treatment, four courses in total. The non-acupressure wrist-ankle strap compression device is shown in Fig. 6B.

After the patients are determined to participate in the study, a trained person will be responsible for guiding upper 1. Daily attendance on the smartphone app will be adopted to ensure that participants wear it every day. At the end of each course, the wearing condition of the strap will be followed up by telephone.

In addition, all patients will be given CBTI. CBTI has achieved widespread scientific recognition as an effective treatment for a wide variety of insomnias. This approach can effectively correct the wrong sleep cognition and inappropriate behavior factors of patients with insomnia [19]. The study adopts the treatment proposed by Michael L. Perlis in *Cognitive-behavioral treatment of insomnia: A session-by-session guide*, including stimulus control therapy, sleep restriction therapy, sleep hygiene education, and cognitive therapy [20]. The treatment is conducted systematically and requires therapist-patient dialogues every week (face-to-face or telephone-based dialogues), eight times in total, as a course of treatment. CBTI will be implemented by two professional therapists trained in clinical sleep medicine programs and had psychological qualifications.

### Outcomes

**Fig. 7:**
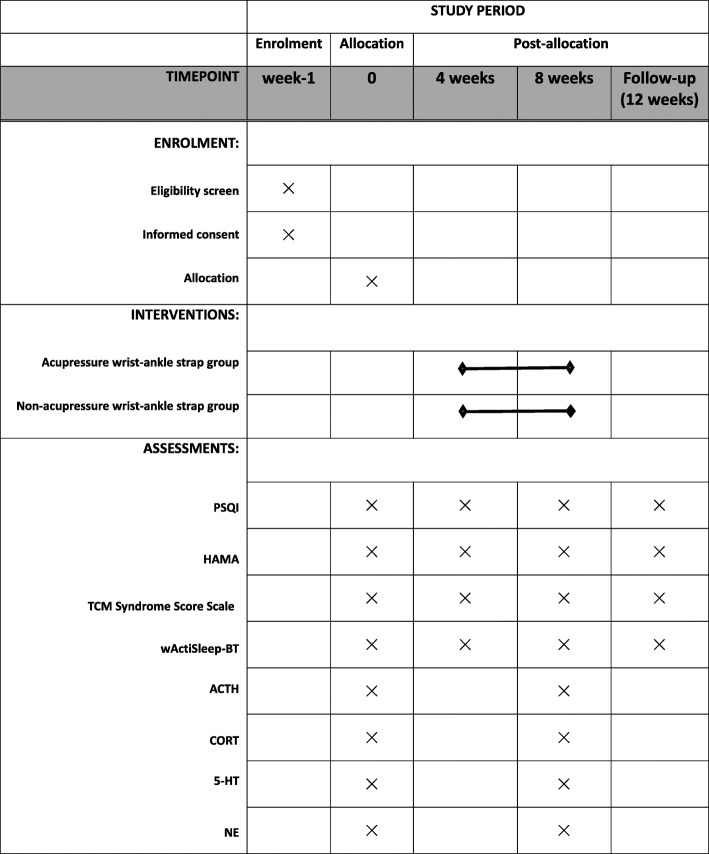
The schedule of enrolments, allocation, and assessments

The schedule of enrolment, interventions, and assessments is shown in Fig. 7. The following outcomes for all the patients will be assessed in person by blinded and independent assessors.

#### Primary outcome

The primary outcome is the Pittsburgh sleep quality index (PSQI) global score, which evaluates a patient's general sleep quality in the past month. The PSQI consists of 19 self-assessment questions and 5 questions assessed by sleep partners. Only 19 self-assessment questions will be scored. The 19 self-assessment questions constitute seven factors from 0 to 3 points. The cumulative score of each factor component is the total score of the PSQI. The total score ranges from 0 to 21. A higher score means worse sleep quality [21]. The PSQI will be assessed at baseline, 4, 8 weeks after randomization and follow-up period.

#### Secondary outcomes

##### PSQI component scores

The PSQI component scores include seven domains, using 0, 1, 2, and 3 points to evaluate the patient's duration of sleep, sleep disturbance, sleep-onset latency, daytime dysfunction due to night sleep, sleep efficiency, need for medications to sleep, and overall sleep quality [17], which will be assessed at baseline, 4, 8 weeks after randomization and follow-up period.

##### Hamilton Anxiety Scale (HAMA)

The HAMA is a commonly used scale in psychiatric clinics, including 14 items. As an important diagnostic tool for anxiety disorders, it is often used clinically as a basis for the diagnosis and degree classification of anxiety disorders. According to the relevant data of the scale, if the score is less than 7, there is no anxiety; if the score is more than 7, there may be anxiety; if the score is more than 14, there must be anxiety; if the score exceeds 21 points, there must be evident anxiety; if the total score exceeds 29 points, it may be severe anxiety. If the scale score is more than 14, it can be diagnosed as an anxiety disorder [18]. HAMA will be examined jointly by two trained assessors. The method of conversation and observation is adopted. Before the start of the clinical trial, the assessors have received 1-week of scale evaluation training by experts to uniform evaluation terms and standardize the evaluation process. The two assessors score independently, and the mean scores will be taken. HAMA will be assessed at baseline, 4, 8 weeks after randomization and follow-up period.

##### wActiSleep-BT monitor

The Actigraphy is a small-sized wristwatch worn on the patient’s wrist before going to bed. It can monitor sleep quality, such as the latency to fall asleep, total sleep time, number and duration of wake-ups, and sleep efficiency. The main sleep parameters are sleep efficiency (SE), total sleep time (TST), and wake after sleep onset (WASO). It will be assessed at baseline, 4, 8 weeks after randomization and follow-up period. Record the value of three consecutive days for each measurement. Values with significant errors will be discarded and take the average value for comparison.

##### Laboratory tests

The pathogenesis of insomnia is that some substances in the brain (such as neurotransmitters, hormones, etc.) act on the sleep center of the human body under the influence of various factors, leading to the abnormality of the central neurotransmitter, and then affect sleep. There is some evidence [6, 7] that the pathogenesis of insomnia is closely related to the circadian secretion of peripheral blood-related hormones in the hypothalamic-pituitary-adrenal axis (HPA axis). In this study, we will detect the levels of adrenocorticotropic hormone (ACTH), cortisol (CORT), serotonin (5-HT), norepinephrine (NE) before and after treatment in the two groups. Participants will receive blood tests in the clinical laboratory of the hospital (items: ACTH, CORT, 5-HT, NE, examination time: 8 a.m., fasting state, venous blood of upper limb), and blood test report issued within five working days by the hospital. The blood tests will be measured at baseline and the 8th week. The laboratory test report will be kept and recorded by an independent data manager.

##### Traditional Chinese Medicine (TCM) Syndrome Score Scale

Syndrome is the objective basis of syndrome differentiation and treatment in TCM. The subjects of this study are mild insomnia patients with anxiety disorders, so the TCM syndrome score scale includes the insomnia part and depression part, the sum of the two is the total score. The syndrome scale with the characteristics of TCM theory is necessary to realize the standardization of TCM syndrome diagnosis and evaluation.

Except for laboratory tests, the primary and other secondary outcomes are detected at baseline and every four weeks. Laboratory data are tested at baseline and the 8^th^ week.

### Statistical analysis

Participants will complete relevant questionnaires and laboratory tests at the first visit. Researchers will guide to ensure the reliability and validity of the questionnaire; meanwhile, a sleep monitor will be completed before the treatment.

All observation results will be documented in the clinical observation forms. Missing data will be replaced according to the principle of LOCF (the last observation carried forward). At the same time, a computer database will be established. The obtained data will be input into the computer on the day of observation records. SPSS 21.0 will be used for statistical processing. Measurement data will be summarized by descriptive statistics (mean, standard deviation (SD), median and interquartile), count data use frequency tables (frequencies and percentages). Normal distribution test and homogeneity test of variance are performed on measurement data. The baseline characteristics of the two groups will be analyzed using independent samples *t* test or Mann-Whitney *U* test (if the distribution was not normal or the variance not homogeneous). If the baseline characteristics are imbalanced, covariance analysis is used to adjust the data. We will use an independent sample *t* test for comparison of between-group comparison and paired sample *t* test for comparison within each group after data meeting normal distribution test and homogeneity test of variance, otherwise using Mann-Whitney *U* test and Wilcoxon signed-rank test. The Pearson chi-square will be performed to compare count data. All statistical tests will be conducted at a 2-tailed significance level of 5%.

### Sample size

PASS 11 software (NCSS, Kaysville, UT) was used to calculate the required sample size. The sample size calculation formula is as follows:

$$ {n}_t={n}_c=\frac{{\left({Z}_{1-\raisebox{1ex}{$\alpha $}\!\left/ \!\raisebox{-1ex}{$2$}\right.}+{Z}_{1-\beta}\right)}^2{s}^2\left(1+\raisebox{1ex}{$1$}\!\left/ \!\raisebox{-1ex}{$k$}\right.\right)}{{\left({\mu}_t-{\mu}_c\right)}^2} $$

Sample size calculation is based on the primary outcome, which is proposed to be the PSQI. Based on the preliminary experiments, the PSQI aggregate score of the acupressure wrist-ankle strap group was 6.125±2.93, and the PSQI aggregate score of the control group was 8.625±3.77. Two groups were 8 cases respectively. The combined standard deviation of the two groups was calculated to be 3.60. That is, in the formula, *μ*_*t*_=6.125, *μ*_*c*_=8.625, *k*=1, and *s*=3.60. In this study, *α*=0.05 and 1-*β*=0.9, therefore $$ {Z}_{1-\raisebox{1ex}{$\alpha $}\!\left/ \!\raisebox{-1ex}{$2$}\right.}= $$1.96, *Z*_1 − *β*_=1.28. Forty five patients are required for each group. Fifty-seven patients will be enrolled in each group after considering an estimated 20% dropout rate, giving a total of 114 patients.

### Trial and data monitoring

The data will be recorded on the paper case report form by a certain assessor and doubled-entered into the electronic case report form. The data and Safety Monitoring Committee of our hospital will monitor the data and review the progress of the trial every three months. Monitors will check the implementation of the study protocol, the treatment of the subjects, and the completion of the informed consent documents every three months. The revision of the plan will be tracked and dated so that the new version can be submitted to the committee.

### Safety evaluation

The acupressure wrist-ankle strap is pure physical therapy, which is safe. Patients need to pay attention not to wear it too tightly or for too long. After wearing it for about 30 min, take it off or loosen it to prevent pressure from causing poor local blood circulation. If the skin is itchy and rash occurs after wearing it, it may be allergic. Patients will be told to stop using the strap and be arranged for a dermatologist. If insomnia worsens after multiple treatments, the patient will be referred to the Department of Neurology for other forms of treatment.

## Discussion

To the best of our knowledge, this is the first registered clinical randomized controlled study of acupressure wrist-ankle straps in the treatment of insomnia. The wrist-ankle acupuncture (WAA) is a unique acupuncture method, which is mainly embodied in the theory of selecting acupuncture points according to body partitions and the subcutaneous superficial needling operation that does not achieve the *de-qi* sensation. According to the basic principles of WAA, each side of the body is divided into six longitudinal zones, and one point is assigned to each of the six longitudinal zones of the wrist and ankle, with the same name as the zone; each point can treat the diseases of the longitudinal zone with the same name. According to the principle of point selection of WAA, insomnia is a symptom that cannot be located. When doing wrist-ankle acupuncture, we choose upper 1 on both sides [22], and so we chose bilateral upper 1 as the points for intervention. The needling points of WAA are distributed on the meridians, and upper 1 is on the Heart Meridian of Hand-Shaoyin. According to the theory of heart storing spirit in traditional Chinese medicine (TCM), insomnia is related to heart dysfunction, and sleep problems can be regulated through the heart meridian [23]. Therefore, the theory of WAA provides a theoretical basis for the ability of the acupressure wrist-ankle strap to improve sleep quality.

In the acupressure wrist-ankle strap group, the compression component of the wrist-ankle strap compresses upper 1 to produce an acupuncture-like effect. Based on WAA theory, the acupressure wrist-ankle strap is a portable WAA point compression treatment strap that treats diseases by automatically applying pressure to acupuncture points, which patients themselves can operate. The therapy is pure physical therapy, non-invasive, painless, non-toxic and side-effects, environment-friendly and low-carbon, recyclable, can be adjusted by people, and is easy to operate. It remains to be verified whether the acupressure wrist-ankle strap and WAA have similarities in the treatment of insomnia. Through the study on the therapeutic effect of acupressure wrist-ankle strap, a new physical intervention method can be added for insomnia, which can be used to relieve insomnia at any time without the intervention of doctors.

In this trial, the patients with mild insomnia prefer physical therapy rather than medication, and there is no high risk without other treatments. Therefore, it is ethically possible for us to include a control group that receives just CBTI. This trial is a rigorously designed randomized controlled, double-blind trial. The design of the non-acupressure wrist-ankle strap, the wrist-ankle strap instruction manual, and follow-up appointments ensure the accuracy of the blind method of this trial. To ensure the quality of this research, before the research officially starts, we conducted a pre-experiment first, consult experts and literature to optimize the test plan based on the pre-test situation, and submitted the study protocol to the medical ethics committee for review and approval; each stage of the trial has an independent researcher to supervise the subject and collect feedback in time; all the research participants, including researchers, receptionists, and data processors, will be trained to ensure the smooth implementation of the trial.

The study aims to provide evidence-based medical evidence for the prevention and treatment of insomnia by acupressure wrist-ankle strap and promotes WAA clinical application. The result of this trial is expected to confirm that the new type of acupressure wrist-ankle strap can significantly improve the sleep quality and anxiety disorder of patients, reduce the suffering of patients, and improve the quality of life; it can also shorten the latency to fall asleep, increase the total sleep time, reduce the number of arousals and duration, improve sleep efficiency; it can reduce ACTH, CORT, 5-HT, and NA in the peripheral blood of patients with insomnia, thereby improving sleep quality.

In view of the above considerations, this study intends to sort out and summarize the clinical data of the new type of acupressure wrist-ankle strap for mild insomnia patients with anxiety disorder in this research group and form a standardized treatment plan. A strictly designed randomized controlled study method will be used to compare the improvement of acupressure wrist-ankle strap and non-acupressure wrist-ankle strap on the patients, to verify its efficacy further and explore the possible mechanism of action, to enrich the treatment of insomnia, improve its clinical efficacy, and lay the foundation for the popularization and application of the acupressure wrist-ankle strap.

The limitation of this trial is that the subjects we will observe are only patients with mild insomnia, and it is not clear whether the wrist-ankle straps are effective for patients with moderate to severe insomnia. This limitation is because the control group uses the non-acupressure wrist-ankle straps, and the whole trial period is eight weeks. Considering that the patients who used to take hypnotics may aggravate the disease after stopping taking the drugs, which will affect their physical and mental health, so only the patients with mild illness will be included. The efficacy of acupressure wrist-ankle straps in patients with moderate to severe insomnia will be verified in the future.

## Trial status

Participants are currently being recruited into the study. The trial began recruitment on 24 October 2020 and will be finished on 31 June 2022.

## Data Availability

Not applicable.

## Abbreviations

BAP: The British Psychopharmacological Association
WAA: Wrist-ankle acupuncture
HPA: Hypothalamic-pituitary-adrenal
CRH: Corticotrophin releasing hormone
ACTH: Adreno-cortico-tropic-hormone
CORT: Glucocorticoids and cortisol
5-HT: Serotonin
NE: Norepinephrine
CBTI: Cognitive behavioral therapy for insomnia
CSMC: Chinese Sleep Medicine Congress
CONSORT: Consolidated Standards of Reporting Trials
PSQI: Pittsburgh Sleep Quality Index
HAMA: Hamilton Anxiety Scale
